# Methods for Gastrointestinal Endoscopy Quantification: A Focus on Hands and Fingers Kinematics

**DOI:** 10.3390/s22239253

**Published:** 2022-11-28

**Authors:** Iván Otero-González, Manuel Caeiro-Rodríguez, Antonio Rodriguez-D’Jesus

**Affiliations:** 1 atlanTTic Research Center for Telecommunication Technologies, Universidade de Vigo, Campus-Universitario S/N, 36312 Vigo, Spain; 2 Hospital Álvaro Cunqueiro, 36312 Vigo, Spain

**Keywords:** quantification, kinematics, smart gloves

## Abstract

Gastrointestinal endoscopy is a complex procedure requiring the mastery of several competencies and skills. This procedure is in increasing demand, but there exist important management and ethical issues regarding the training of new endoscopists. Nowadays, this requires the direct involvement of real patients and a high chance of the endoscopists themselves suffering from musculoskeletal conditions. Colonoscopy quantification can be useful for improving these two issues. This paper reviews the literature regarding efforts to quantify gastrointestinal procedures and focuses on the capture of hand and finger kinematics. Current technologies to support the capture of data from hand and finger movements are analyzed and tested, considering smart gloves and vision-based solutions. Manus VR Prime II and Stretch Sense MoCap reveal the main problems with smart gloves related to the adaptation of the gloves to different hand sizes and comfortability. Regarding vision-based solutions, Vero Vicon cameras show the main problem in gastrointestinal procedure scenarios: occlusion. In both cases, calibration and data interoperability are also key issues that limit possible applications. In conclusion, new advances are needed to quantify hand and finger kinematics in an appropriate way to support further developments.

## 1. Introduction

Quantification is the act of counting and measuring that maps human sensory observations and experiences into quantities. It is fundamental to the scientific method as it produces a standardized form of measurement that allows the application of statistical procedures involving not just values of the observed phenomena but also metrics such as accuracy, precision, or reliability.

In recent years, quantification has been facilitated by advances in sensor technology to a considerable extent. Motion capture is a specific type of quantification that involves the capture of data regarding the movements performed by a person or an object. Recently, new vision-based systems, such as the Microsoft Kinect (MS Kinect) system for Windows, have provided a quick, cheap, and easy way of analyzing position and mapping three-dimensional (3D) pose data, providing skeletal movement tracking. The availability of easy-to-use quantified solutions has facilitated the application of these technologies in many areas. Regarding motion capture, human body movements related to the practice of sports such as tennis, football, running, hockey, basketball, swimming, or even walking in high-heeled shoes have been quantified to support the assessment, training and improvement of movements [1]. In the area of gait analysis, it has been demonstrated that wearable sensors can replace laboratory systems [2]. Similarly, wearable sensors can also be used to quantify physiological levels and provide indicators of health or physiological features, such as stress [3] or mood [4].

This paper is focused on the quantification of Gastrointestinal (GI) endoscopy procedures. Currently, lower GI endoscopy, also known as a colonoscopy, is the gold standard procedure for the screening, diagnosis and treatment of lower GI pathologies, and the demand for GI endoscopies has increased dramatically [5]. Nevertheless, performing GI endoscopies involves some important challenges. Firstly, a colonoscopy is a complex procedure that requires skilled operators to perform difficult manual maneuvers with very good hand–visual coordination [6]. Secondly, a high number of procedures (275–500) is generally required before an acceptable competency is acquired by an endoscopist [7], [8] and existing competency assessment methods are based on subjective observation [9]. Thirdly, the workload of existing endoscopy units is often high, with the need to perform an increasing number of endoscopies while supervising, training, and instructing future endoscopists [10]. Fourthly, colonoscopy training is performed with real patients that can suffer discomfort, inconvenience, or even be harmed during the procedures by trainees [11]. Finally, endoscopists themselves are exposed to musculoskeletal conditions (injuries, numbness, and pain) in different parts of their bodies (back, neck, shoulders, elbows, hands/fingers) because of factors such as [12,13,14,15] high force, endoscopic maneuvers requiring repeated pinching, uncomfortable postures, prolonged awkward movements, etc. The quantification of endoscopist movements and other related features can be a useful approach for understanding these challenges, supporting the assessment training and improvement of endoscopists.

The goal of this paper is to explore the available technological solutions to quantify GI endoscopy procedures, particularly focusing on endoscopist hand and finger movements. In recent years, several research papers have been published related to the quantification of GI endoscopy procedures. These are reviewed in Section 2, involving features such as the force exerted on the endoscope, endoscopist and endoscope movements, eye gaze, etc. Surprisingly, none of these works involve the quantification of hand and finger motion, despite the fact that they seem to be key for the performance of appropriate endoscope maneuvers in a safe and efficient way. Section 3 is focused on the methods to quantify hand and finger kinematics, considering the possible movements and the current technologies, both vision-based and sensor–sensor. Next, Section 4 introduces an experimentation setting to assess these technologies with specific devices, smart gloves and cameras for tracking. Section 5 describes the results of this experiment and the advantages and drawbacks of the different devices. In Section 6, we discuss the problems detected and contribute possible solutions. The paper finishes with some conclusions in Section 7.

## 2. Related work

Table 1 shows solutions explored to quantify GI endoscopy procedures.

### 2.1. Direct Observation

Before quantification, GI endoscopy procedures have already been measured based on direct observation. Indeed, there exist several competence measurement proposals based on expert observations [34], such as the Gastrointestinal Endoscopy Competence Assessment Tool (GiECAT), Global Assessment of Gastrointestinal Endoscopy Skills (GAGES), Assessment of Competency in Endoscopy (ACE), Direct Observation of Procedural Skills (DOPS), etc. To a significant extent, they are focused on the manipulation of the endoscope controls, particularly considering hands and fingers.

Already in 2002, [16] colonoscopy procedures were recorded in video and analyzed by experts to asses endoscopist skills [16], focusing on instrument handling by the endoscopist, the endoscopic luminal view, and a continuous display of the colonoscope configuration using a magnetic endoscope imaging. Hand and finger position and movements are important in the scales, where they are considered to assess skills, such as (i) manipulation of instrument controls including grip, tip/steering control, and the adequacy of suction/insufflation/irrigation; (ii) manipulation of the insertion tube, including insertion tube grip, rotation, and attempts to straighten the colonoscope; and (iii) depth of insertion.

A similar experience in 2009 describes the application of the OCcupational Repetitive Action (OCRA) method using videos of recorded procedures [17]. The main concern, in this case, was the high prevalence of musculoskeletal disorders affecting endoscopists. To this end, they analyzed the risk of upper limb biomechanical overload. This method evaluates four main collective risk factors based on respective duration: repetitiveness, force, awkward posture and movements, and lack of proper recovery periods.

Direct Observation of Procedural Skills (DOPS) is a Workplace-Based Assessment (WBA) instrument that has been applied to colonoscopy procedures [35]. This has been used to evidence the quality of the clinical competence of colonoscopy practitioners. According to [10], DOPS assesses the following items related to the basic handling of the colonoscope: “grip of instrument with accurate finger/thumb”, “control of wheels”, “tip steering”, and “manipulation of the shaft”. The DOPS metric “incorrect use of hand grip” was found to be one of the most significant ones, showing improvement with a week of intensive training. The Competency Model for Skills Training in GI Endoscopy in Ireland [36] is another DOPS proposal that includes a more detailed description of hand and finger skills to be observed:

- Scope handling: (1) Exhibits good control of the head and shaft of colonoscope at all times; and (2) Demonstrates ability to use all scope functions (buttons/biopsy channel) whilst maintaining a stable hold on the colonoscope. Minimizes external looping in the shaft of the instrument.
- Tip control: (1) Integrated technique, as the ability to combine tip and torque steering to accurately control the tip of the colonoscope and maneuver the tip in the correct direction; and (2) Individual components: tip steering, torque steering and luminal awareness.
- Air management: Appropriate insufflation and suction of air to minimize over-distension of the bowel while maintaining adequate views.
- Loop management: Uses appropriate techniques (tip and torque steering, withdrawal, position change) to minimize and prevent loop formation.

### 2.2. Pressure and Force Measurement

Several authors have developed experiments to measure the pressure and force exerted by the endoscopist during the performance of colonoscopy procedures:

- Shergill et al. (2009) evaluated upper-extremity musculoskeletal load [15]. Particularly, they measured right-thumb pinch and downward force and bilateral forearm-muscle activity in both left and right forearms. Essentially, they found high left wrist extensor muscle activity and high right thumb pinch force loads exceeding the threshold levels known to increase the risk of injury [18]. An extension of the previous experiments was performed more recently in 2021, collecting data from thumb pinch force and forearm muscle loads [18]. Differences between male and female endoscopists were observed at the biomechanical level.
- Ref. [6] A wireless device called the Colonoscopy Force Monitor (CFM) was used to measure the manipulation patterns and force applied by the hands and fingers during the insertion and withdrawal of the tube during a colonoscopy [6,19]. This device was able to capture the over the endoscope: linear force (push/pull or axial) and radial force (torque). [26] A sensor on the hose of the colonoscope was also used in [26] for the real-time measurement of the force and torque of the colonoscope applied by the hands and fingers. This work was not focused on training or injury prevention but on supporting the endoscopist by providing information about the force and posture of the distal end of the endoscope to avoid bowel perforation and looping. They refer to previous designs of the endoscope, identifying some issues, particularly that they are bulky and demand two-handed operation.

### 2.3. Motion Quantification

Motion quantification in endoscope procedures has considered two different objects: endoscope and endoscopist. Regarding endoscope movements, there are several studies [20,22,25,31,33] that attempt to quantify features such as longitudinal and circular displacement of the endoscope along the larger intestine, tip angulation, angular velocity, and rotation [22,25,31].

Regarding endoscopist movements, there are also several authors that have already attempted to quantify them:

- In [23,24], right wrist posture and movements were analyzed using a magnetic motion-tracking device. Endoscopists wore a right arm sleeve and glove that were custom-made for this study. The [24] hypothesis of these studies was that the range of wrist movement (mid, center, extreme, and out) for each wrist DoF (flexion/extension, abduction/adduction, pronation/supination) might decrease as experience in colonoscopy is acquired. It was concluded that fellows spent significantly less time in an extreme range of wrist movements at the end of the study compared to the baseline evaluation.
- Microsoft Kinect^TM^ was used in [10] to measure the technical skills of endoscopists. Seven metrics were analyzed to find discriminative motion patterns between novice and experienced endoscopists: hand distance from the gurney, the number of times the right hand was used to control the small wheel of the colonoscope, the angulation of the elbows, the position of the hands in relation to body posture, the angulation of the body posture in relation to the anus, the mean distance between the hands, and the percentage of time the hands were approximated to each other.
- In Ref. [28], a motion tracking setup to measure wrist and elbow joint motions is described in [28]. Several wrist and elbow motion metrics are described in this work. For each wrist, the axes of flexion/extension and abduction/adduction motions were analyzed. For each elbow, the axes of flexion/extension and supination/pronation movements were analyzed. For each joint, the number of times the joint entered extreme ranges of motion, as well as the total time spent in extreme ranges of motion.
- In Ref. [29], flexible wearable sensors that were placed on the dorsum of both hands and the dorsal section of both forearms (2/3 distance from wrist to elbow) were used in [29,30] to compare differences in the movements between novices and experts [30]. Three-dimensional coordinates from the length of the endoscope were taken using a Magnetic Endoscopy Imaging system called ScopeGuide (UPD-3, Olympus, Tokyo, Japan) that includes electromagnetic coils along the length of the endoscope.

### 2.4. Other Quantifications

In Ref. [32], endoscopy eye gaze during simulated colonoscopy procedures was quantified [32]. This study is relevant because, during an endoscopic procedure, the endoscopist needs to manipulate the scope by coordinating the video images on the monitor with his/her hand movements. It is quite common for the endoscopist to become disoriented because of a loss of both global and local spatial references when navigating the scope inside the bowel. Differences between novices and experts were observed during the analysis of the data.

In Ref. [21], force and positional quantification were measured together. Considering the limited endoscopic maneuvers, the integration of measurements of force and posture sensors with the observation of video recordings was explored in [21]. Similarly, in [27], the use of [27] several tracking systems was described: a wireless motion sensor system to track the endoscopist’s body, a magnetic probe inserted in the working channel of the endoscope to track the endoscope, a webcam to track the endoscope wheel’s rotation, and an external camera to record the procedure.

## 3. Hand and Finger Kinematics Quantification

### 3.1. Hand and Finger Kinematics

**Figure 1:**
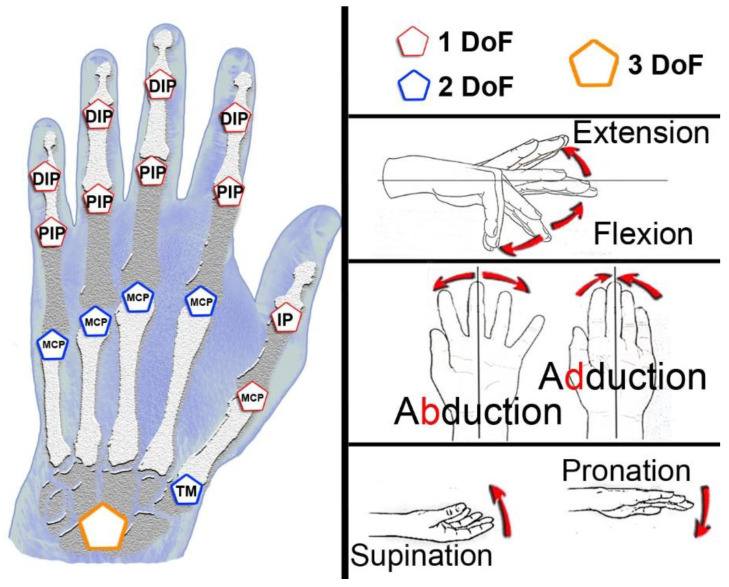
Representation of degrees of freedom in wrist and fingers.

The distribution of hand movements is, in most cases, a combination of several simple movements. To know in detail the rectilinear movements, Degrees of Freedom (DoF) were proposed for the study as the equivalent of these movements in each of the joints in both directions but within a straight line. In this way, the set of movements that a healthy person can perform is detailed below (See Figure 1). The flexion/extension movements are represented with a hexagon with a red outline that symbolizes 1 DoF. The movements are symbolized with a blue hexagon that adds to the red hexagon symbol of the abduction/adduction movement, adding 1 DoF. They are, therefore, movements that have 2 DoF. Finally, the orange hexagon symbolizes the set of movements of the wrist. This encompasses the 2 DoF explained above and added the movement of pronation/supination, which comes from the elbow. The orange hexagon represents 3 DoF. The total movements of the hand are included in this scheme to represent the 23 DoF of the hand. From the fingertips to the wrist, we find three phalanges on each finger (distal, intermediate, and proximal); only the thumb has two (distal and proximal). The joints that join the bones are the distal interphalangeal (DIP), proximal interphalangeal (PIP), and metacarpophalangeal (MCP). In the unique case of the thumb, these are the interphalangeal (IP) and metacarpophalangeal (MCP). There is another joint to consider inside the hand that is called the trapeziometacarpal (TM) (See Figure 1, left side). Finally, in the unique case of the wrist, 3 DoF have been identified for simplicity since the wrist is a joint composed not only of a set of bones and joints, but we must also consider the structure of the ligaments that form it [37]. In the case studies, some DoFs are not performed independently. For example, many participants cannot perform distal finger flexion without interphalangeal finger flexion.

### 3.2. Quantification Technologies

Measuring and evaluating the movements of fingers and hands is, therefore, a vital process to determine the future steps that will help us correct and prevent future injuries [38]. To this end, there are methodologies and proposals for measuring the movement capabilities of the hands and upper limbs, such as (UEFT) [39], Sollerman [40], and Wolf [41]. In addition, there are already commercial products whose intention is to record the biomechanical information of the fingers and hands [42].

#### 3.2.1. Sensor-Based Technology

Sensors can be used in isolation or integrated into a glove to identify movements of interest, such as joint rotations. The use of sensors solves the occlusion problem, and the biomechanical information is displayed in real-time [43]. The comfort in this case of endoscopy is measured in the ability of the gloves to be the least invasive on the sensitive actions that the specialist himself performs. Another advantage of gloves is that the data collected is direct, unlike cameras, where the registered data set must be subsequently processed and modeled to identify which joints and, therefore, which movements are performed.

#### 3.2.2. Vision-Based Technology

This consists of a set of cameras that record the movement of markers that are in a predefined volume with the aim of identifying the trajectory and position with great precision, among others. For these cases, occlusion is a factor that can be reduced with more cameras, favoring triangulation and estimating the position, as well as the optimization of trajectory-reconstruction algorithms [20,21].

### 3.3. Smart Gloves

Smart gloves are devices that incorporate features that allow them to be used in different fields, both industrial and research. In addition, commercial and academic interest in them is growing. In a recent review of current commercial smart gloves that we have conducted [42], we reviewed the technologies involved, the main applications, and the current state of development. Most smart gloves are wireless devices that use Bluetooth or WiFi communication and are composed of actuating elements that can transmit tactile and kinesthetic information. When data transmission is based on a wired connection, this advantage of ubiquity and freedom is lost. Gloves, rings, wristbands, and watches can serve as an always-on interface between events and triggers within the virtual environment and physical space. In some cases, integrated sensors can provide tracking or movement information. Due to low-capacity batteries, they are unable to record long sessions.

These devices are really very useful in providing a real-time sensation of interaction in a virtual setting, but currently, the uncomfortable design reminds the user that they are wearing the gloves. In this case, although the experience is close to reality, it prevents the user’s brain from assuming that they are making natural movements in a virtual environment similar to real life where these gloves are not in use [44]. Feedback is more real with wearable haptic devices despite their 500 g weight. The application of these smart gloves has been successfully applied to human–computer interactions and other fields, which play an increasingly important role [42]. At present, because most smart gloves use hard sensors, the comfort of these gloves has been poor [45]. The objective in these cases is the use of flexible sensors that were quickly integrated into these smart gloves, improving this issue [46,47]. Below are two examples of the most popular gloves, showing the evolution from smart gloves that use rigid sensors, Manus VR Prime II, to smart gloves that use flexible sensors, MoCap Pro Gloves.

#### 3.3.1. Manus VR Prime II

**Figure 2:**
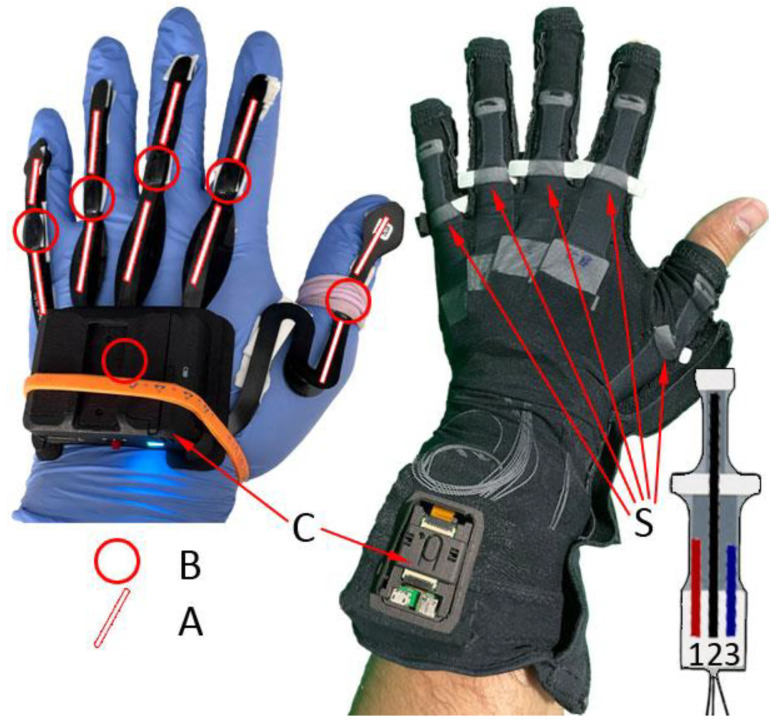
Distribution of the sensors in the Manus Prime II VR gloves (left) and the MoCap Pro Gloves (right). A—IMU; B—Rigid sensors; C—Bluetooth communication controller; S—Stretch sensors that measure the bend and splay of each finger.

This device consists of a glove made of a synthetic fiber fabric that adapts to the hand with different sizes (See Figure 2, left). Manus Prime II VR is made of five flexible sensors and six IMUs. It has a communication system for the transmission of the data captured by both the cable and Bluetooth. This glove measures 19 DoF, which translates into the capacity to measure the movements that the hand performs, for example, for each finger and the wrist. The flexion and (hyper) extension of each MCP and wrist joint are analyzed. The flexion and (hyper) extension of the thumb TM joint are analyzed. The flexion of each PIP joint is analyzed. The abduction and adduction of each MCP are analyzed. The flexion of the DIP joints is estimated on the individual flexion of each IP joint. The pronation and supination of the wrist are analyzed. The abduction and adduction of the wrist are analzyed.

#### 3.3.2. MoCap Pro

This device consists of a glove made of elastic nylon fabric that adapts to the hand (See Figure 2, right). MoCap Pro is made of five stretch sensors, each one with three channels of communication (Ver Figure 2C), and one accelerometer sensor in the wrist position. It has several sizes and a communication system for transmitting the data captured both by the cable and Bluetooth. In addition, it has the option for SD memory storage and, therefore, allows the data to be extracted later.

### 3.4. Camera-Based Systems

Computer vision (CV) methods are often used to trace arms, hands, or fingers. Its use is highly recommended since it often does not require entering or using elements that the user must wear. As for the hands, the most common application is gesture recognition and posture estimation. Both are very challenging due to the complex structure and dexterous movement of the human hand, which has 23 degrees of freedom (DOF). There is a large body of literature on gesture-tracking methods that employ CV-based methods. Rautaray and Agrawal [48,49] studied CV-based hand-gesture recognition for HCI. Chen et al. [50] examined hand-gesture recognition using 3D depth sensing and 3D hand-gesture recognition approaches. They also undertook research on methods based on deep learning. Vuleti et al. [51] conducted a review of the hand gestures used in HCI. Chen et al. [52] conducted a comprehensive and timely review of the real-time sensing and modeling of human hands with wearable sensors or CV-based methods. Alam et al. [53] provided a comprehensive survey on intelligent voice and vision applications using deep neural networks. Beddiar et al. [49] reviewed and summarized the progress of human activity recognition systems from the perspective of machine vision.

Vero Vicon. v2.2

**Figure 3:**
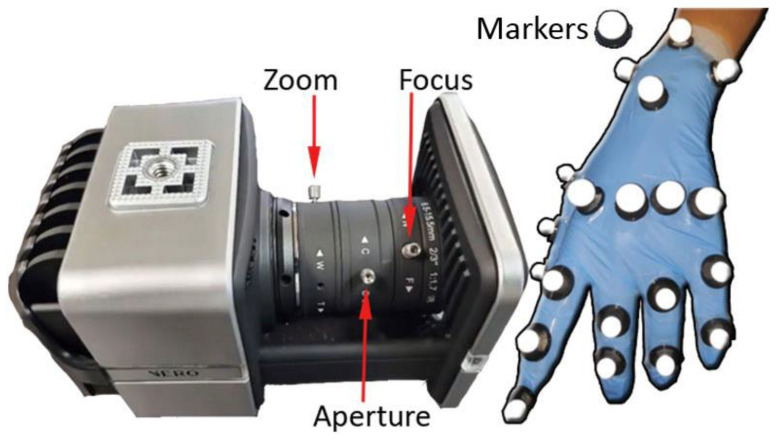
Main functionalities of Vero Vicon and example of distribution of markers in the hand.

Optical tracking systems, such as the VICON system (see Figure 3, left), are primarily used to capture full-body movements such as gait but are also used for the detailed capturing of hand movements, as by Lee and Tsai [36] with only six markers, which were able to recognize up to 20 static Taiwanese Sign Language hand gestures with high accuracy. The VICON camera tracks reflective markers that are attached to different parts of the hand, mainly the back. The use of six cameras means that it is possible to avoid the problem of occlusion in many cases; in our case, the occlusion is caused using endoscopic control. VICON’s accuracy for marker tracking is 1 mm, providing us with very accurate data for tracking and mapping. Figure 3 shows an example of a configuration in the placement of markers on the hand (See Figure 3, right).

This VICON 3D motion capture system consists of six Vero 2.2 cameras, 2.2 Megapixels with a 6–12 mm varifocal lens and an IR stroboscope. Each camera has a resolution of 2048 × 1088, that is, 2.2 mpx at 330 fps, which allows for the capture of fast movements such as sports, even with multiple actors with very low latency. It contains a built-in accelerometer that notifies the user when a recalibration is needed. Each camera has its own RJ45/Cat5e cable to connect directly to the PC, so the session can be run safely and independently of any individual issues.

## 4. Experimentation

This section explains the process of performing a colonoscopy and simulating it on a mannequin.

This section is divided into three subsections: (i) the motion capture systems, (ii) the colonoscopy process, and (iii) the aspects to be evaluated in the two proposed capture systems.

### 4.1. Motion Capture Devices

Two types of systems were tested, and they are described in Section 3. The first system involves smart gloves that consist of IMUs and other sensors for the measurement of finger position and to obtain hand and finger tracking: Manus VR Prime II and MoCap Pro smart gloves. The other system is camera-based, consisting of optical tracking technology: Vero Vicon v2.2 system.

Although the vision system is generally used to monitor the body, head and eyes, in this case, it has focused on monitoring the movement of some markers placed on the hand and fingers (see Figure 3, right) in order to obtain the tracking and modeling of the hand thanks to its accuracy for subsequent data processing. These devices were chosen because they are both affordable options for capturing motion data during colonoscopies, as well as being compatible with each other through their use of infrared LEDs and cameras for measuring movement.

### 4.2. The Colonoscopy Process

The colonoscopy process involves the insertion of a flexible tube into the anus and then through the rectum and large intestine (colon) [54]. This is performed by a doctor or a technician. The aim of the colonoscopy is to detect cancerous polyps and remove them if they are found. Furthermore, this procedure is an examination that allows visualizing the entire large intestine and the final part of the small intestine (terminal ileum) [55].

**Figure 4:**
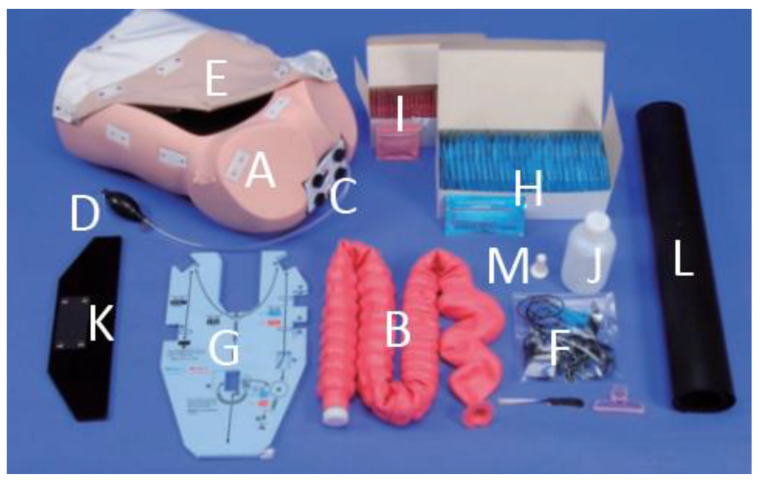
Components of the dummy for the experiment. A—Abdomen model; B—Colon–rectum tube; C—Anus unit; D—Air bulb; E—Abdomen skin cover; F—Colon tube fixtures; G—Colon layout guide; H—Concentrated lubricant, blue pack; I—Endoscope lubricant gel, pink pack; J—Plastic bottle; K—Stand; L—Rubber sheet; M—Anus plug.

The research work considers the training space that is located inside the Meixoeiro public hospital in the city of Vigo. Here, there is a practice room that has the necessary instrumentation and even a simulation kit with a dummy to carry out colonoscopy practices. This room tries to reproduce a scenario very similar to the one that the specialist can find during a real intervention, where both beginners and specialists meet for their first contact or to apply new techniques. In this practice room, a dummy stands out, as can be seen in the image (see Figure 4) [56]. It represents the thoracic and abdominal parts of a human being. A template is placed inside (See Figure 4, G), indicating the exact placement of the plastic digestive tube (See Figure 4, B). The kit has 10 templates that allow the user to gradually increase the difficulty of the practice. The soft, flexible colon tube provides a realistic response to using a colonoscopy for skills such as preventing “loops’’ and safely reaching the cecum. The colonic tube can be made airtight, allowing for insufflation and the suctioning of air. The dummy can be oriented in the left lateral, right lateral, or supine positions.

The learning techniques that can be applied in this scenario consist of carrying out the complete exploration procedure until reaching the cecum, the tube extraction process, and detailed observation of the walls with 360° turns to observe all the folds that are found along the gut cavity, among others. These practical exercises are much more complex in a real intervention because there are fluids that make vision difficult and, in addition, the uniqueness of each patient, which makes mobility through the intestinal cavity a challenge. However, temperance and tranquility are acquired with experience, which is an advantage that allows specialists to solve unforeseen events that add human risk [57].

### 4.3. Aspects Evaluated of the Two Proposed Capture Systems

The main aspects of assessing these two types of devices are, on the one hand, technical and, on the other, ergonomic. We must bear in mind that having this type of device requires a very high initial investment with an unpredictable amortization of its use. In addition, in the case of gloves, they are experimental products whose support and survival are in the hands of startups and companies with a very short life in the market, between 3 and 4 years. Therefore, an important factor is the knowledge and experience of the company with similar products. The direct contact with providers via webinars gave us the confidence to trust these proposed products.

Therefore, ignoring high costs and having confidence in the providers, we have divided fundamental aspects into two:

Technical aspects:

- Installation (complicated/time/limited license);
- Calibration (more or less complex and fragile);
- Battery (limitation in measurements/Autonomy);
- Communication (Bluetooth or cable);

Ergonomic aspects;

- Ease of use (difficulty due to gender or hand size);
- Naturalness (naturalness of use during the handling of tools).

There are two types of aspects that are relevant when taking measurements in a doubly complex medical procedure. On the one hand, the medical technique itself is considered one of the most complex medical techniques, especially when it requires the use of electrical cuts or the removal of cancerous layers on the lining of the intestine. On the other hand, there is the recording and monitoring of hand movements, on which there are few studies focused on obtaining good and bad mobility of the fingers and hands.

In this scenario of practice with a mannequin where there is less pressure than what can exist with an intervention in a real patient, the basic exercises that are proposed to come into contact with the endoscope in an imaginary colonoscopy are: turn the camera, left–right up–down; turn left–right big and small wheel; insert and extract hose with right hand.

## 5. Results

### 5.1. Set Up Process

This section shows the result of the processes that are necessary to start the selected devices to measure hand and finger movements during colonoscopy practices. A series of previous actions must be considered in the computer where the processing, capture, and communication with the devices are centralized. Among them, the installation of the software itself stands out, as well as the updating of both the work interface and the treatment of the data received. In addition, in the case of Smart Gloves, firmware updates are also performed. A process that must be carried out by cable and that must be repeated for each one, that is, for both the right and left glove in both glove models.

These are innovative devices that are in a constant process of change both at the hardware and software levels. To start up these commercial devices, whose objective is to use them for measurement, the following steps are needed: installation and update of the device’s own software, acknowledgement of the license acquired, connection to devices, device update, calibration, data capture, and data treatment.

The results obtained that allow us to compare the installation and calibration times that are important in these devices are shown in Table 2.

Contrary to glove-based devices, whose initial calibration takes less than 1 min, the average time of 90 min for the Vero Vicon calibration stands out. This long time is needed for the calibration and adaptation of the cameras in order to obtain the greatest sharpness in the capture of the markers. These markers must be positioned so that at least three of the cameras always have direct vision to triangulate their position. When this does not happen, a gap is produced, which is due to the fact that during a number of frames, there has been an occlusion on the marker. Furthermore, it is important to point out the high sensitivity of these devices, which would require the individual calibration of one of the cameras or of the set.

#### 5.1.1. Facility

During the first installation step, generally, each device has a guide in which we are invited to download the latest version from its official website. This application, which must generally be running in the background, is essential to be able to use any of the devices. During this step, the license provided during the purchase process is required to activate the program, which can be requested before or after the download process.

#### 5.1.2. Connection

The connection interface between the glove or the cameras is established with the computer, normally by cable, as is the case with the Vero Vicon, which is connected by means of an RJ45 Ethernet cable. However, although this option is allowed with gloves, it is much more comfortable and practical with a wireless connection. This communication is possible thanks to a dongle, which is a small device that is connected to the computer to provide an additional function, which in this case is the transmission of data to a model recreated in the program that is integrated into the initial installation.

#### 5.1.3. Calibration

Once we have verified the status of the gloves or the cameras and that these devices both appear visually on the equipment screen with all the indicators ready, we proceed to the calibration process. In the case of the gloves, the calibration steps consist of imitating a series of proposed poses and maintaining that position for a few seconds. In this way, the calibration is saved in the glove associated with the person who uses it. In the case of cameras, the process is much more complex. It consists of visualizing the markers, which in this case will be distributed throughout the hand (See Figure 4, right), which is achieved by adjusting the zoom, focus, and aperture parameters (See Figure 4, left) and thus achieving good focus and definition. In addition, other elements must be prevented from producing noise, such as reflections. As a final step, a series of movements are performed with a specific cane that has a pre-designed distribution of markers for effective recognition of the movement of the cane. This more tedious calibration process in Vero Vicon is due in part to the fact that the final position of the cameras may require modification for the optimal setup. The cameras must capture the volume in which the previously placed markers are going to move so that each marker can be viewed by at least three cameras. In this way, for each marker, we can always know its position.

#### 5.1.4. Data Treatment

The common type of data recorded by all devices are quaternion data [58]. The atomic measure of the quaternion could be collected for each joint or each marker in the case of Vero Vicon. A quaternion can represent a 3D rotation and is defined by four real numbers. x, y, and z represent a vector, and w is a scalar that stores the rotation around the vector. The most accurate data are obtained with Vero Vicon, with an error of 0.1 mm [59]. However, the error estimate is not defined for either glove. Although with all devices, the data are recorded and extracted directly, the steps to obtain valuable information to evaluate some characteristics of the movements are not direct. It is necessary to use external programs such as Open Sim [60]. An opensource software package that allows users to develop models of musculoskeletal structures and create dynamic simulations of movement. In the case of the hands, there are no models, so this is a future challenge to be achieved as a contribution to the community.

### 5.2. Lessons Learned with the Devices

In this second section of the results, the experiences that have been collected during the measurements with the different devices are explained. The purpose is to make known aspects such as the naturalness of use during the handling of tools as well as their independence in terms of autonomy.

#### 5.2.1. Manus VR Prime II Gloves

##### Experienced Inconveniences

- Rigid gloves prevent the natural mobility of the hand and therefore are more uncomfortable.
- One size in the purchase, which means having several units.
- Does not allow raw data to be extracted directly for further analysis.
- Does not exchange finger movement data with other measurement devices such as Xsens. To do that, the Prime Xsens version is necessary.

##### Advantages and Benefits Experienced

- Relatively good battery capacity, allowing 40 min of measurement.
- Wireless enables remote work provided by Bluetooth.
- Support for incorporating external position trackers.
- Data recorded on own encrypted format manus
- Data could be exported as FBX

#### 5.2.2. Stretchsense MoCap Pro Gloves

##### Experienced Inconveniences

- Little adaptable to smaller hands, for this specific case, the fingertips are covered, which is inconvenient for the specialist.
- Calibration by individual poses, slower process than with Manus, which is more sequential.
- Difficulty putting on gloves, requiring help from a partner.
- Adduction abduction movements are not appreciated and therefore are not clearly reflected in the hand model.
- The bar graph that shows the movements is very small and does not allow us to appreciate the variation of the data.

##### Advantages and Benefits Experienced

- Very flexible and therefore comfortable to the touch, allows for more natural measurements.
- Approximately 3 h of use at full capacity.
- Offers alternatives for transmission and/or storage of data captured with SD memory.
- Allows you to extract the captured raw data directly in csv format, quaternion output.

#### 5.2.3. Vero Vicon

##### Experienced Inconveniences

- Very delicate calibration regarding the sensitivity of the cameras.
- Assembly time and preparation of the capture scenario of more than 1 h.
- Raw data captured in its own session format and not directly analyzable.
- Requires a prior study of marker placement for later modeling and data interpretation.
- The markers are the essential elements to monitor, and sometimes they do not allow for a natural movement of fingers and hand in certain exercises.

##### Advantages and Benefits Experienced

- Unlimited battery capacity.
- Very high precision in data capture with a maximum average error of 1 mm.
- Allows you to extract raw data in csv, quaternion output.
- Allows you to extract vrpn, pose data automatically supplied. VRPN clients that are able to make use of velocity and acceleration data can use this information directly from Tracker’s VRPN output rather than having to calculate this on the client side. This is useful if, for example, you want to use Vicon data within a dead reckoning algorithm or other prediction algorithms to estimate poses at a time that may or may not coincide with a Vicon frame.

Other factors of interest regarding desirable future improvements and detected problems can be found in the next section.

## 6. Discussion

This research work has been proposed with the aim of evaluating the technologies that would allow us to quantify the movements and positions of the hands in a singular case of the use of endoscopy techniques. A study of injuries such as Quervain’s tenosynovitis, tennis elbow, neck and back pain [61], among others. Injuries are produced by the repetitive manipulation of the endoscope and with a poorly ergonomic endoscope, especially for small hands [62]. However, due to the many difficulties described in this work, we have been forced to do a preliminary task of identifying and evaluating the experiences gained during the measurements made with two types of devices. Among the difficulties, we have found, apart from those described in Section 5.2, the following:

- None of the analyzed devices offer integration for the analysis of these movements and their correlation with musculoskeletal injuries.
- The two types of gloves studied do not offer a precision range, which calls into question the validity of the data for the scientific study and the quality of the results.
- The markers distributed in this case by hand cause two important problems: (i) the occlusion that causes gaps and, therefore, a loss of information about its trajectory and position (ii) and the difficulty of manipulating the tool, making the maneuver difficult.
- There are very few studies that focus their studies on the analysis of hand modeling due in part to the fact that it is an extremely complex part of the body.

Despite the limitations and problems discussed, the authors of this work are optimistic about the evolution and improvement of these devices, whose interest has increased within the scientific community due to their application in several areas of study in recent years, as reported in previous works [42]. They also offer some proposals that are considered the future challenges of this research, such as:

- Make data transmission compatible in both directions so that the precision of the cameras is used to correct a problem they have with occlusions. In addition, the analysis and post-processing of data from both points of view, that of the hands and body posture, would allow for obtaining more significant conclusions.
- Design personalized gloves that are highly adapted to the contour and silhouette of the hand, not interfering and not hindering the fine manipulation of microsensors such as Polhemus Micro Sensors. Therefore, the design of a glove with an array of infrared LEDs is proposed, just as the Vero Vicon calibration stick is installed.
- Modeling of the hand integrated with Deep Learning so that it speeds up and corrects the gaps that are produced by the occlusion of the markers or, failing that, the array of infrared LEDs that integrates the previously proposed glove.
- Standardized data analysis that integrates data types such as quaternion, which are recorded by these devices avoiding intermediate filtering and pre-processing for subsequent reproduction and simulation of mobility. A simulation is already offered by free software programs such as openSim, which is recognized by the scientific community.

## 7. Conclusions

Efforts to track hands in the total set of studies analyzed are a small part of scientific interest. However, there are great advantages that a meticulous and detailed study would entail in certain work areas in which handling a tool is the main part of the execution of a task, such as a colonoscopy case study. There is particular interest in devices such as smart gloves that can be integrated into applications such as video games, military, or rehabilitation, but their introduction for the monitoring of movements and, therefore, the improvement in their execution with the capture of good and bad practices has not been found, as far as we know, until the publication of this article. The fact of making an effort to publicize the previous phases of setting up and handling all of the devices analyzed is of internal utility, and thus it has been understood that it is to make it known to the scientific community. It ensures us that despite the necessary previous steps in capturing with a Vero Vicon camera system, which can be long and tedious, it is a very good investment of time. It ensures the quality and precision in the capture of movements and makes calibration between participants unnecessary, giving specialists a perception to be measured immediately.

It should be noted that the time of these specialists is very valuable. The advantage offered by the gloves is the flexibility in the location to measure using them since a fixed space prior to assembly is not required, in addition to a wireless measurement. The interest of the authors goes beyond this exposition of the devices with their characteristics as well as the lessons learned from their use. As future and complementary works, the intention is to continue the study focusing on the data obtained and their relevance for the study of movements.

## Figures and Tables

**Table 1:** Publications about quantification related to the performance of gastrointestinal endoscopy procedures (“col.” stands for “colonoscopy”).

Publication	Measurement Method/Devices	Entity	What Is Measured	Participants and Procedures
[16] Shah et al., 2002	Observations (in video)	Upper limbs	Manipulation skills of endoscope controls. Endoscope insertion tube manipulation. Depth of insertion	18 endoscopists 22 colonoscopies
[17] Battevi et al., 2009	Observation (in video)	Upper limbs	Frequency of technical actions per minute. The awkward posture of the hand continuously moving in a pinch of the endoscopic wheels. For the left arm, the static posture to bear instruments. Fine movements of left-hand fingers. Right elbow flexion–extension movements. Wrist postures in ulnar deviation of the right thumb.	6 endoscopists 4 gastroscopies 4 colonoscopies
[15] Shergill et al., 2009 [18] Shergill et al., 2021	Pressure: tactile pad Force: surface EMG	Right thumb Right and left forearm bilateral muscles	Right thumb pinch force Forearms muscle activity	6 endoscopists 30 colonoscopies
				7 experienced, 9 trainees Total: 49 real col.
[6] Korman et al., 2010 [19] Korman et al., 2012	Pressure: load cells	Endoscope tube	Push/pull force Torque: clockwise and counterclockwise	3 experienced endoscopists 9 real col.
				12 endoscopists. 2–4 real col.
[20] Obstein et al., 2011	Kinematics: electromagnetic 6D motion sensors	Endoscope tube	Path length, flex, velocity, acceleration, jerk, tip angulation, angular velocity, rotation and curvature of the endoscope. A kinematic score is calculated for the endoscopists	9 novice endoscopists 4 experienced ones A physically simulated col.
[21] Browne and O’Sullivan, 2012	Kinematics: electrogoniometers Force: surface EMG	Wrist Dominant forearm	- Wrist posture (flexion/extension) - Muscle activity	10 novice endoscopists 5 (physically simulated stomach wall biopsy)
[22] Arnold et al., 2013	Kinematics: motion sensors	Endoscope tube	Longitudinal displacement Circular displacement	25 colonoscopies
[10] Svendsen and Hillingsø., 2014	Kinematics: Microsoft Kinect TM	Body	Level of the left hand below the *z*-axis. Level of the right hand below the *z*-axis. Distance between hands. Distance shoulder-hand. Angulation of right elbow. Angulation of left elbow. Angulation of shoulders to the anus	10 experienced 11 novices A physically simulated col.
[23] Mohankumar et al., 2014 [24] Ratuapli et al., 2015	Kinematics: magnetic tracker (Polhemus Fastrak)	Wrist	Wrist posture (flexion/extension, abduction/adduction, pronation/supination) Wrist motion range (mid, center, extreme, and out) for each wrist DoF (flexion/extension, abduction/adduction, pronation/supination)	12 endoscopists 2 VR simulated col. 5 first-year fellows 4 VR simulated col.
[25] Nerup et al., 2015	Magnetic endoscope imaging	Endoscope tube	Colonoscopy Progression Score (CoPS) A map of progression in colonoscopy	11 novices. 10 experienced A physically simulated col.
[26] Zheng et al., 2017	Force: resistance-strain	Endoscope tube	Axial force: push and pull force Torque: clockwise and counterclockwise	-
[27] Velden, 2018	Kinematics: IMU trackers (Xsens) Kinematics: magnetic Webcam and color band External camera- Kinematics: Vision (M.Kinect 2)	Body Endoscope tube End. wheel rotation Endoscopic view	23 body segments: pelvis, L5, L3, T12, T8, neck, head, right and left shoulder, upper arm, forearm, hand upper leg, lower leg, foot, and toe. Translation and torque. Angle of the two endoscope wheels Insertion, retroflexion and inspection, duodenal intubation, and retraction Head	9 endoscopists (3 beginners and 6 experts) 26 uppers gastrointestinal procedures
[28] Holden et al., 2018	Kinematics: electromagnetic 3D motion sensors	Hand, arm and forearm (wrist and elbow joints)	The total path length of hands, number of discrete hand motions, wrist motions (flexion/extension, abduction/adduction) elbow motions (flexion/extension, supination/pronation), number of times in extreme ranges of motion.	22 novice, 8 experienced A physically simulated col. (Wooden bench-top model)
[29] Levine et al., 2018 [30] Krill et al., 2019	Kinematics: motion trackers (Biostamp RCTM)	Left and right forearms Forehead	3D linear accelerations Angular velocity	19 gastroenterologists A VR simulated col.
				30 novices, 18 experienced
[31] Vilmann et al., 2020	Kinematics: motion sensor	Endoscope tube	3D data: travel length, tip progression, chase efficiency, shaft movement without tip progression, looping	12 novices, 12 experienced 2 physicals simulated col.
[32] He et al., 2020	Eye gaze tracking (Tobii X2-60)	Eye gaze	Scope disorientation. Eye gaze fixation. Eye gaze saccade	20 novices, 6 experienced VR simulated
[33] Fulton et al., 2021	Kinematics: magnetic tracker (Polhemus Patriot)	Endoscope tube	Translation trajectory and rotation of the endoscope	Physically simulated col.

**Table 2:** Comparison of the installation and setting up characteristics.

Name	Software Installation Time ^1^	Communication Type	Initial Calibration Time	Session Calibration Time ^1^	Calibration Time between Participants
Manus VR Prime II	30 min	1 dongle	<1 min	<2 min	<2 min
Mocap Pro Gloves	30 min	2 dongles	<1 min	<5 min	<2 min
Vero Vicon v2.2	50 min	6 RJ45 wires	90 min ^2^	<5 min	0 min

^1^ Keep in mind that installation times depend a lot on the computer you are working with, for example the number of processors and memory. ^2^ The number of cameras to be used together with the object of study to be measured can vary the times. In this case, 6 cameras are used.

## Data Availability

Data sharing is not applicable to this article.
